# Enhancing Health Benefits through Chlorophylls and Chlorophyll-Rich Agro-Food: A Comprehensive Review

**DOI:** 10.3390/molecules28145344

**Published:** 2023-07-11

**Authors:** Tânia Martins, Ana Novo Barros, Eduardo Rosa, Luís Antunes

**Affiliations:** 1 Centre for the Research and Technology of Agro-Environmental and Biological Sciences, University of Trás-os-Montes and Alto Douro (CITAB), 5000-801 Vila Real, Portugal; taniam@utad.pt (T.M.); erosa@utad.pt (E.R.); labanimalsgroup@gmail.com (L.A.); 2 Institute for Innovation, Capacity Building and Sustainability of Agri-Food Production (Inov4Agro), 5000-801 Vila Real, Portugal

**Keywords:** chlorophylls, chlorophyllin, health, biological activity

## Abstract

Chlorophylls play a crucial role in photosynthesis and are abundantly found in green fruits and vegetables that form an integral part of our diet. Although limited, existing studies suggest that these photosynthetic pigments and their derivatives possess therapeutic properties. These bioactive molecules exhibit a wide range of beneficial effects, including antioxidant, antimutagenic, antigenotoxic, anti-cancer, and anti-obesogenic activities. However, it is unfortunate that leafy materials and fruit peels often go to waste in the food supply chain, contributing to the prevailing issue of food waste in modern societies. Nevertheless, these overlooked materials contain valuable bioactive compounds, including chlorophylls, which offer significant health benefits. Consequently, exploring the potential of these discarded resources, such as utilizing them as functional food ingredients, aligns with the principles of a circular economy and presents exciting opportunities for exploitation.

## 1. Introduction

**Figure 1:**
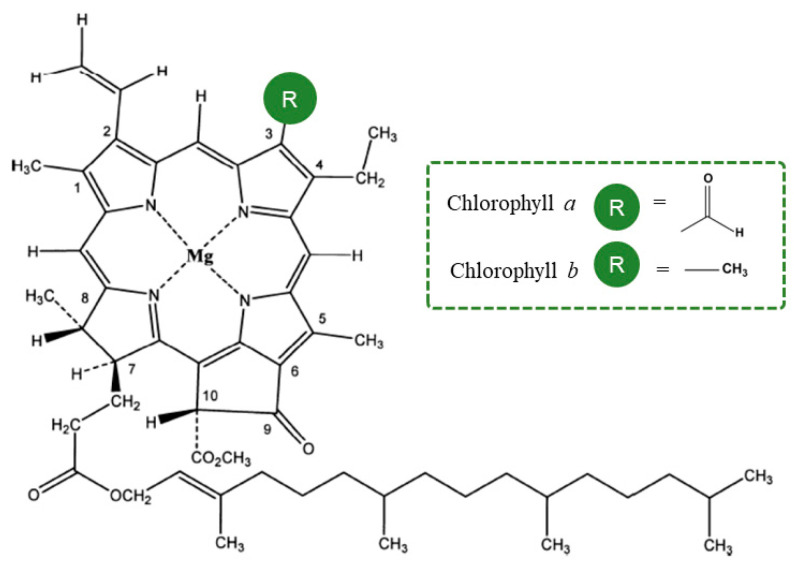
Structure from Chlorophyll *a* and Chlorophyll *b*.

Chlorophyll, Figure 1, a complex green pigment found in plants, algae, and certain bacteria, plays a crucial role in the process of photosynthesis by absorbing light energy and converting it into chemical energy [1]. While early beliefs about the bioavailability and stability of chlorophyll under differaent conditions limited research on its health effects, recent studies have shed light on the potential benefits of chlorophyllin as a chemopreventive agent [2]. Nagini et al. have provided insights into its molecular mechanisms [2]. Although in vitro and in vivo studies suggest its anticancer effects, evidence of its efficacy in humans remains scarce [2]. Dietary supplements containing chlorophyll and chlorophyllin are available and generally considered safe, with no reported adverse side effects over several decades of human use [3]. However, skepticism about their effectiveness persists due to the lack of robust scientific evidence supporting their claimed health benefits [3].

Despite the potential health benefits associated with chlorophyll, a significant number of chlorophyll-rich vegetables, leafy materials, and fruits are lost throughout the food supply chain [4]. This loss occurs despite the underutilized potential of these agro-food residues [5,6]. Harnessing and utilizing this discarded material could contribute to the transition towards a more sustainable circular economy.

Due to the poor bioavailability and stability of chlorophylls, studies on chlorophylls are scarce until now. In summary, the main purpose of this review is to provide a comprehensive understanding of the current knowledge on chlorophylls and their potential applications in the context of bioavailability, stability, antioxidant activity, and antimutagenic properties. The results and conclusions can be applied by readers and researchers to deepen their knowledge, guide future studies, explore applications in food and pharmaceutical industries, and stimulate further research in this field.

## 2. Chlorophyll: Chemical Properties and Metabolism

Chlorophyll is a complex molecule made up of a porphyrin ring, a magnesium ion, and an attached hydrocarbon tail. The porphyrin ring is responsible for absorbing light energy and the magnesium ion acts as an electron acceptor. Chlorophyll has many forms such as chlorophyll *a*, chlorophyll *b*, chlorophyll *c*, chlorophyll *d* and chlorophyll *e* [1].

The most common form of chlorophyll found in plants is chlorophyll *a*. Its chemical structure includes a porphyrin ring with a central magnesium ion, and an attached hydrocarbon tail known as a phytol. The porphyrin ring is made up of four nitrogen-containing groups called pyrrole, and the phytol tail is composed of isoprenoid units [7]. Chlorophyll *a* absorbs light most efficiently in the red and blue regions of the spectrum, with peak absorption at around 430 and 662 nanometers, respectively.

Chlorophyll *b* is another form of chlorophyll found in plants, algae, and some bacteria. Its chemical structure is similar to that of chlorophyll *a*, but it has a slightly different porphyrin ring. This difference results in chlorophyll b absorbing light in the blue-green region of the spectrum, with peak absorption at around 453 nanometers. Chlorophyll *b* also has a role in photosynthesis, but its main function is to protect chlorophyll *a* from excess light. In addition to chlorophyll *a* and *b*, there are other forms of chlorophyll such as chlorophyll *c*, chlorophyll *d*, and chlorophyll *e*. These are found in a variety of organisms such as algae, and they have different absorption spectra and different functions. Chlorophyll *c* absorbs light in the blue-green region of the spectrum, chlorophyll *d* absorbs light in the red region of the spectrum, and chlorophyll *e* absorbs light in the far-red region of the spectrum [7].

Photosynthesis is the process by which plants, algae, and some bacteria convert light energy into chemical energy. The light energy is absorbed by chlorophyll and other pigments, which excite electrons in the pigment molecules. After absorbing light energy, the excited electrons in chlorophyll are utilized to facilitate the synthesis of ATP (adenosine triphosphate) and NADPH (nicotinamide adenine dinucleotide phosphate), which are essential components for the subsequent phases of photosynthesis. These energy-rich molecules play a crucial role in the production of glucose and the release of oxygen as byproducts. The first stage of photosynthesis is known as the light-dependent reactions which take place in the thylakoid membrane of chloroplasts. The light energy absorbed by chlorophyll and other pigments is used to drive the transfer of electrons, which results in the production of ATP and NADPH. The second stage of photosynthesis is known as the light-independent reactions, also called the Calvin cycle, which takes place in the stroma of chloroplasts. In this stage, the ATP and NADPH produced in the light-dependent reactions are used to drive the production of glucose and oxygen [8].

Chlorophyll, chlorophyllides, and phycobiliproteins such as phycoerythrin and phycocyanin, are all pigments that are involved in the process of photosynthesis. Chlorophyll is the primary pigment found in plants and algae, while chlorophyllides and phycobiliproteins are found in smaller quantities. The main difference between these pigments is their chemical structure, which results in different absorption spectra and therefore different functions in photosynthesis.

The process of obtaining pheophorbides from chlorophyll is called chlorophyll degradation, which is a process that occurs naturally in plants and algae. This process can be triggered by different environmental factors such as light intensity, temperature, and water availability. During chlorophyll degradation, chlorophyll is broken down into different pigments, including pheophytin, which is a form of chlorophyll that lacks a magnesium ion, and pheophorbide, which is a form of chlorophyll that has been modified by the removal of the phytol tail [8].

**Figure 2:**
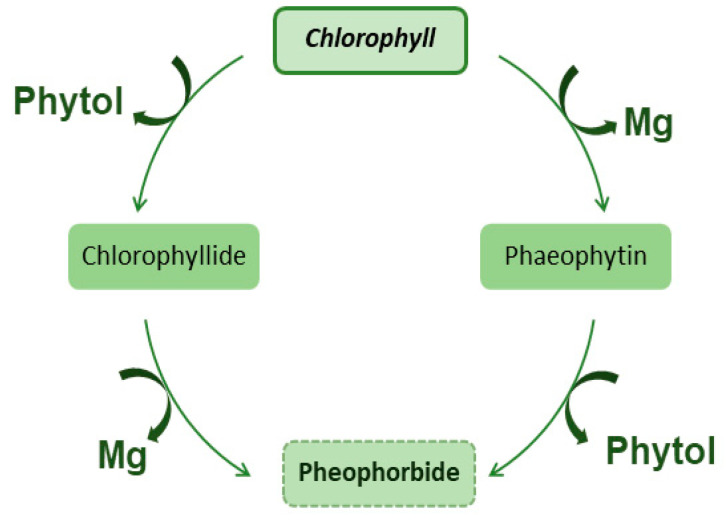
Conversion of Chlorophyll in phaeophytin, chlorophyllide and pheophorbide.

Chlorophyllides are pigments that are closely related to chlorophyll, and they differ in the arrangement of atoms. Their chemical structure is similar to chlorophyll but they have a different central atom such as zinc, iron, or copper, and they have different absorption spectra. They are found in prokaryotic organisms such as cyanobacteria and they have a role in photosynthesis similar to chlorophyll [9], Figure 2.

Phycobiliproteins such as phycoerythrin and phycocyanin are found in cyanobacteria and red algae. They are water-soluble pigments that are composed of a protein component and a pigment component. They absorb light in different regions of the spectrum than chlorophyll, they transfer the energy to chlorophyll and they are important in photosynthesis by increasing the efficiency of light harvesting [10].

## 3. Effects of Chlorophyll in Health

### 3.1. Historic Perspective/Herbal Ethnomedicines

The utilization of medicinal plants has a long history, dating back to ancient times, with its practice spanning across different regions worldwide [11,12]. Even in the present era, herbal ethnomedicines continue to be of fundamental importance in primary healthcare, particularly for impoverished populations in remote areas [13] and developing countries [14]. These medicinal plants are employed to address a wide array of illnesses, including cancer, skin diseases, cardiovascular disorders, endocrinal imbalances, gastrointestinal ailments, genitourinary conditions, respiratory system disorders, musculoskeletal disorders, liver diseases, and even treatment for poisonous bites, among others [12,13,15,16,17,18]. While the therapeutic effects of medicinal plants are often attributed to their secondary metabolites [18,19,20,21], it is worth noting that photosynthetic pigments may also play a significant role [5,6], with chlorophyll being the most well-known pigment associated with these plants.

Among the various plant parts used for ethnomedicinal purposes, leaves stand out as the most utilized [22,23,24,25,26]. Leaves possess several advantages, such as easy accessibility, straightforward processing, prolonged availability, and therapeutic properties resulting from the accumulation of photosynthates and phytochemicals [18,20,24,27]. Notably, harvesting leaves does not pose a threat to the survival of the plant, unlike the harvesting of whole plants or roots, which can endanger the survival of medicinal plant species and contribute to a decline in plant biodiversity within specific regions [22,23].

### 3.2. Chlorophyll Bioavailability

Chlorophylls, the most abundant pigments on Earth, are present in photosynthetic organisms such as bacteria, algae, and higher plants. In plants, chlorophyll a and b are the predominant pigments, with their ratio varying depending on the species, environmental conditions, and ripening stage [6]. However, chlorophylls are highly sensitive to physical and chemical changes and exhibit instability when isolated or consumed outside of their biological context. This has led to the development of chlorophyll derivatives [28]. Consequently, natural chlorophylls are not commonly used in experimental research, as their purification is challenging and expensive.

Semi-synthetic sodium copper-chlorophyllins (SCC) have been commercially available as food colorants and supplements. SCC is produced through the saponification of natural chlorophylls, where the magnesium ion in the tetrapyrrole is substituted by copper and the hydrophobic side chain is eliminated. These modifications enhance the stability, solubility in water, and accessibility of SCC [29,30]. Commercial SCC products may vary in composition, although two primary components, Cu-chlorin e4 and Cu-chlorin e6, are typically present [31,32,33]. Due to these advantageous properties, SCC has found widespread use in biological experiments. However, it is important to note that the bioavailability of SCC may not match that of natural chlorophylls obtained from our diet.

Chlorophylls can be obtained from the human diet through the consumption of green fruits and vegetables. However, their content varies significantly depending on factors such as cultivar, maturity stage, growing conditions, harvest time, plant parts used, storage conditions, food processing methods, and extraction and quantification techniques [6,28]. While a diet rich in vegetables and green fruits may provide a substantial amount of chlorophylls, their bioavailability, metabolism, and the effects of food processing influence their potential impact on human health. Early studies assumed that humans did not absorb chlorophylls, resulting in limited research on their absorption through the gastrointestinal tract. However, a few studies have demonstrated that native chlorophylls undergo significant transformation during the digestive process, and the absorption of different chlorophyll derivatives may differ based on their molecular structure.

In vitro studies have provided valuable insights into the digestion, metabolism, and absorption of natural chlorophylls. Using an in vitro model simulating the gastric and small intestinal digestive processes, Ferruzi et al. [34] demonstrated that native chlorophylls obtained from fresh spinach puree undergo various transformations. The highly acidic gastric phase led to the conversion of chlorophylls into their metal-free pheophytin derivatives, while Zn-pheophytins treated with ZnCl_2_ remained stable. Furthermore, the micellarization of chlorophyll a series was found to be more efficient compared to the b series. The uptake of micellarized chlorophyll derivatives by Caco-2 human intestinal cells predominantly consisted of pheophytins and their epimers, comprising around 5–10% of the absorbed compounds. Another study utilizing the same in vitro approach revealed that Cu-chlorin e4 in SCC remained stable during digestion, whereas 90% of Cu-chlorin e6 underwent degradation. However, incorporating SCC into a food matrix reduced the degradation of Cu-chlorin e6 [32]. Additionally, SCC derivatives were taken up by Caco-2 cells and transported to the basolateral compartment, suggesting their potential absorption and transport to peripheral tissues [32,34].

Furthermore, a study using chlorophylls from pea puree demonstrated that native chlorophylls were completely transformed into their magnesium-free derivatives during gastric digestion *in vitro*, primarily due to the acidic conditions [35]. Pheophorbide a, the most micellarized chlorophyll derivative, exhibited the highest absorption by Caco-2 cells [35]. Another investigation by Gandul-Rojas et al. [36], using standard pigments such as chlorophyll a, chlorophyll b, pheophytin a, pheophytin b, pyropheophytin a, pheophorbide a, and pyropheophorbide a, indicated that the de-esterification of the phytol group enhanced the transfer efficiency of dephytylated derivatives (pheophorbide a and pyropheophorbide a) to the aqueous micellar fraction and their transport to Caco-2 intestinal cells, thereby increasing their bioaccessibility. Moreover, the cellular uptake of phytylated chlorophyll derivatives (pheophytin a and pyropheophytin a) by Caco-2 cells occurred through simple diffusion, whereas the uptake of dephytylated derivatives was mediated by facilitated diffusion at lower concentrations tested [36].

A recent study investigated the in vitro digestion of chlorophyll pigments derived from three types of edible dried seaweeds: Nori (red algae, containing only chlorophyll a series), Sea lettuce (green algae, containing a and b series), and Kombu (brown algae, presenting a and c series). The findings revealed that chlorophylls from a series were more susceptible to pheophytinization compared to the b and c series. The formation of pheophorbides during digestion occurred when the initial chlorophyll profile contained significant amounts of pheophytins, as observed in the Kombu algae. Furthermore, the digestive stability and recovery of chlorophyll derivatives after in vitro digestion appeared to depend on the chemical structure and the food matrix [37]. The same authors demonstrated that the percentage of micellization and the uptake by Caco-2 cells were higher for dephytylated chlorophylls compared to phytylated derivatives. Additionally, chlorophylls from Nori algae exhibited higher bioaccessibility than those from Sea lettuce and Kombu [38].

Regarding studies demonstrating in vivo absorption of chlorophyll derivatives, a chemoprevention trial in humans using SCC revealed that daily ingestion of SCC tablets (300 mg/day) resulted in the absorption of Cu-chlorin e4 ethyl ester into the bloodstream, and to a lesser extent, Cu-chlorin e4 [31]. This provided the initial evidence that chlorophyll derivatives can be absorbed by the human gastrointestinal tract. Another study conducted on human volunteers detected the presence of pheophytin and pheophorbide derivatives in the blood three hours after the ingestion of 1.2 kg of freshly boiled spinach [39]. In a study by Gomes et al. [33], rats fed a diet supplemented with 10 or 30 g/kg SCC showed absorption of Cu-chlorin e4, which was detected in the serum, liver, and kidneys. The absorption was macroscopically visible as a green color in these biological materials. However, Cu-chlorin e6 was not found in the serum or organs, suggesting degradation during passage through the gastrointestinal tract or interaction with other food components [33].

Regarding chlorophylls from natural sources, Fernandes et al. [40] studied the apparent absorption of chlorophylls in dogs by analyzing ingested and excreted chlorophylls. The results showed that after supplementation with 0.8% freeze-dried ground spinach leaves (18 mg chlorophyll/day) for 10 days, the average apparent absorption of chlorophyll derivatives was 3.4%. By analyzing the excreta, the authors inferred that pheophytinization, the major degradation process occurring in the acidic gastrointestinal tract of dogs, transformed chlorophylls a and b into their respective magnesium-free pheophytins a and b. However, they were unable to detect chlorophyll derivatives in the plasma of dogs after consuming a diet containing 10% freeze-dried spinach [40]. In a biodistribution study conducted in rabbits, where a single dose of 100 g of freeze-dried spinach powder (prepared from fresh spinach) was administered after 24 h of fasting, and rabbits were sacrificed after 2, 4, 8, 12, and 24 h, the major metabolites observed were chlorophyllides and pheophorbides [41]. The occurrence and levels of chlorophyll derivatives were organ-specific, found in the plasma, liver, gallbladder, and kidney. In the feces, the major metabolites detected were native chlorophylls and pheophytins [41]. More recently, Vieira et al. [42] analyzed the livers of mice fed a diet containing 15% spirulina powder, a blue-green microalgae primarily composed of chlorophyll a. The results demonstrated that the formation and absorption of pheophorbides, pyro-derivatives, and phytyl-chlorin e6 required first-pass metabolism. The absorption and accumulation of pheophorbide a in the liver may be partially protein-mediated through the scavenger receptor B type I, while the presence of phytol in the liver may occur due to the de-esterification of pheophytin a, leading to the formation of pheophorbide a and phytol [42]. In the feces, the percentage of pheophorbide derivatives and allomerized derivatives was similar to that in the supplemented feed, while pheophytin derivatives and pyro-derivatives exhibited increased content compared to the supplemented feed. Additionally, native chlorophyll a was detected in the feces of mice [42].

In summary, the collective findings from both in vitro and in vivo studies using native chlorophylls indicate that the potential health benefits associated with chlorophyll a and b are likely attributed to their metal-free derivatives. While these studies provide valuable insights into the bioavailability of chlorophylls, there is still a need for a comprehensive characterization of the chlorophyll derivatives formed in the gastrointestinal tract and a better understanding of their pharmacokinetic parameters. Unfortunately, as of now, there is a lack of published in vivo studies involving omnivorous species, which poses challenges in translating this information directly to humans. Further research in this area is warranted to bridge the gap in our understanding of chlorophyll metabolism and absorption in the human body.

### 3.3. Bioactive Properties of Chlorophyll Compounds

The chemical structure of chlorophylls is a key determinant of their bioactivity, influencing their potential health benefits [5]. Understanding the relationship between chemical structure and bioactivity is crucial for unraveling the therapeutic properties of chlorophylls and their derivatives [43,44,45]. The chemical structure of chlorophylls consists of a porphyrin ring, which serves as the core framework, and a long hydrophobic side chain. This unique structure confers distinctive physicochemical and biological properties to chlorophylls. For instance, the presence of magnesium at the center of the porphyrin ring enables chlorophylls to efficiently capture light energy during photosynthesis.

Moreover, the chemical structure of chlorophylls contributes to their bioactive properties. Studies have shown that chlorophyll derivatives, which undergo structural modifications, exhibit enhanced bioactivity compared to native chlorophylls. These modifications can involve alterations in the porphyrin ring, such as the addition of functional groups or substitutions. These structural changes lead to variations in the solubility, stability, and interaction capabilities of chlorophyll compounds [7,46].

The bioactivity of chlorophylls is attributed to their ability to act as antioxidants, antimutagens, and anticarcinogens. The unique chemical structure allows chlorophylls to scavenge harmful free radicals, mitigate DNA damage, and modulate cellular processes involved in disease development. Furthermore, their hydrophobic side chains facilitate interactions with biological membranes, influencing cellular uptake and signaling pathways [5,47].

Despite advances in understanding the relationship between the chemical structure of chlorophyll compounds and their bioactivity, further research is warranted. Investigations into the specific structural features responsible for different bioactive properties are necessary to unlock the full potential of chlorophylls as therapeutic agents. Moreover, exploring the interactions between chlorophylls and other bioactive compounds in a synergistic manner can provide insights into the holistic benefits of consuming chlorophyll-rich foods [48].

**Figure 3:**
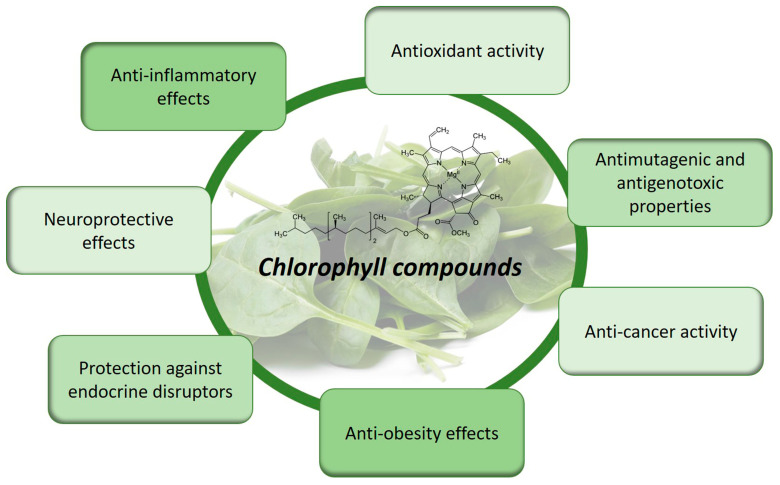
Bioactive properties and health benefits of chlorophyll compounds.

In conclusion, the bioactive properties of chlorophyll compounds are intricately linked to their chemical structure. By deciphering the structural determinants of their bioactivity, we can uncover new opportunities for utilizing chlorophylls and their derivatives in promoting human health [47]. Continued research in this field holds great promise for harnessing the full potential of chlorophylls as functional ingredients and contributing to the development of novel therapeutic approaches (Figure 3) [49,50,51].

#### 3.3.1. Antioxidant Activity

Oxidative stress plays a significant role in the development of various diseases. Natural chlorophylls possess antioxidant properties [52], which make them promising candidates for preventing or mitigating the formation of reactive species. In vitro studies conducted by Ferruzi et al. [32] demonstrated that standard chlorophyll a derivatives exhibited higher antioxidant capacity compared to chlorophyll b derivatives. However, Lanfer-Marquez et al. reported contrasting findings, showing that pheophorbide b and pheophytin b were the most potent natural antioxidant derivatives compared to chlorophyll a derivatives [45]. Ferruzi et al. [32] also observed that metallo-chlorophyll derivatives (such as Mg-chlorophylls, Zn-pheophytins, Zn-pyropheophytins, Cu-pheophytin a, and Cu-chlorophyllins) exhibited higher antiradical capacity than metal-free derivatives (such as chlorins, pheophytins, and pyropheophytins). This suggests that in addition to the fundamental porphyrin structure contributing to free radical reduction [53], metal chelation enhances antioxidant activity [32]. Moreover, Lanfer-Marquez et al. [45] demonstrated that Cu-chlorophyllin displayed higher antioxidant activity compared to natural chlorophylls, highlighting the influence of the chelated metal in the porphyrin ring on the strength of the antioxidant capacity. In an in vitro study conducted by Kang et al., Zn-pheophytins exhibited the highest radical scavenging capacity and β-carotene bleaching activities, surpassing the antioxidant activity of chlorophylls and pheophytins [54]. Zn-pheophytins also demonstrated inhibitory activity against lipopolysaccharide (LPS)-induced nitric oxide production and suppressed the expression of inducible nitric oxide synthase in LPS-stimulated macrophage cells without exerting cytotoxic effects [54]. Regarding in vivo investigations, Gomes et al. [33] reported that a diet supplemented with SCC protected against lipid peroxidation in the brain of rats, but not in the liver. Additionally, pretreatment with chlorophyll b in mice resulted in reduced oxidative stress and lipid peroxidation induced by cisplatin, although the antioxidant effects were inconsistent [55]. Despite the potential antioxidant activity of natural chlorophylls, research in this area has been relatively limited in recent years. This may be attributed to the perception that chlorophylls are less important compared to other phytochemicals found in fruits and vegetables, as well as the belief that chlorophylls are poorly absorbed by the human gastrointestinal tract. However, it is worth noting that the understanding of the antioxidant properties of natural chlorophylls is an evolving field, and further investigations are necessary to fully elucidate their potential and establish their significance relative to other phytochemicals.

#### 3.3.2. Antimutagenic and Antigenotoxic Properties

Mutagenic and genotoxic agents are ubiquitous in our environment and food, and some are even used as chemotherapy agents such as cisplatin. In a pioneering study by Lai et al. [56], the chlorophyll content of fruits and vegetables was correlated with their antimutagenic activity. Using the gene reversion mutagen test with Salmonella strain TA100, the researchers found that higher chlorophyll content in both aqueous and acetone extracts corresponded to decreased mutagenic activity induced by 3-methylcholanthrene or benzo[a]pyrene [56]. Interestingly, the study also revealed that SCC had a more potent effect than vegetable extracts. In another study conducted in *Drosophila melanogaster*, the effects of SCC and natural chlorophylls from spinach and chlorella were evaluated against the genotoxicity of 4-nitroquinoline 1-oxide (4NQO) in the flies [57]. All the chlorophyll preparations demonstrated a reduction in wing spot formation caused by 4NQO. The authors proposed that the mechanisms of action involve the formation of complexes between chlorophyll and 4NQO, as well as the inhibition of metabolic activation of this mutagen through enzyme inhibition or degradation of active metabolites [57]. Similarly, Kocaoğlu Cenkci and Kaya [58] demonstrated the effectiveness of chlorophyll a and b in reducing the genotoxic effects of 2-amino-3,8-dimethylimidazo [4,5-f]quinoxaline in *Drosophila melanogaster*. An additional study evaluated the antimutagenic effect of standard chlorophyll derivatives in *Salmonella typhimurium* TA100 exposed to the mutagen benzo[a]pyrene. The results indicated that both metallo and metal-free derivatives exhibited similar dose-dependent antimutagenic activity, suggesting that the tetrapyrrole macrocycle is essential for chlorophyll’s antimutagenic activity rather than the presence of a central metal atom [32]. In mice, pretreatment with chlorophyll b was found to mitigate chromosomal breakage and micronucleus formation induced by cisplatin in peripheral blood and bone marrow cells [55]. Similar protective effects were observed in the liver and kidneys, where DNA damage was reduced [59]. Despite these promising findings, the potential antimutagenic and antigenotoxic activity of natural chlorophylls remains largely unexplored, similar to their antioxidant activity mentioned earlier. Further research is needed to comprehensively investigate and understand the extent of these properties in natural chlorophylls.

#### 3.3.3. Anticancer Activity

Cancer, as the second leading cause of death worldwide, necessitates the development of new therapeutic agents with minimal side effects. Studies have demonstrated the potential anticarcinogenic action of chlorophylls and sodium copper chlorophyllin (SCC) against various types of cancer. For instance, topical application of pheophorbide a inhibited skin tumor promotion induced by 7,12-dimethylbenz[a]anthracene (DMBA) and 12-O-tetradecanoyl-phorbol-13-acetate (TPA) in ICR mice [60]. Similarly, chlorophyll a and b extracted from green tea leaves suppressed skin tumorigenesis and edema formation in BALB/c mice when applied prior to treatment with the tumor promoter TPA, initiated by DMBA [61].

In rainbow trout exposed to the potent environmental carcinogen dibenzo[a,l]pyrene (DBP), concurrent exposure to native chlorophyll preparations or SCC significantly reduced DBP-DNA adduct levels in the liver [62]. Dietary intake of natural chlorophyll and SCC also reduced DBP-DNA adducts in the liver, inhibited tumor incidence and multiplicity in the liver, and reduced tumor incidence in the stomach of rainbow trout when co-fed with DBP [63,64]. Aflatoxins, potent inducers of hepatocellular carcinoma (HCC), are food contaminants produced by fungi. Chlorophyllin forms a strong noncovalent complex with aflatoxin-B1 (AFB1), inhibiting hepatic AFB1-DNA adduction and hepatocarcinogenesis in rainbow trout [65,66,67]. In rats administered with AFB1, chlorophylls derived from spinach and chlorophyllin protected against early biochemical and late pathophysiologic biomarkers of AFB1 carcinogenesis in the liver and colon [68]. This protection is thought to occur through the inhibition of AFB1 intestinal uptake involving complex formation, thereby reducing its bioavailability [68]. In a study involving human subjects from Qidong, People’s Republic of China, where aflatoxin contamination in food is linked to a high risk of HCC, the consumption of 100 mg of chlorophyllin three times a day for 4 months reduced the urinary excretion of aflatoxin-DNA adducts by 55%, indicating a reduced biologically effective dose of aflatoxin [50].

High consumption of red meat is associated with an increased risk of colon cancer. However, studies in rats subjected to dietary heme, which mimics red meat ingestion, demonstrated that natural chlorophylls inhibit colonic cytotoxicity, proliferation of colonic epithelial cells, epithelial cell turnover, and the formation of lipid radicals induced by heme [69]. In contrast, Na-chlorophyllin and Cu-chlorophyllin were unable to prevent these heme-induced effects [69]. Diets supplemented with freeze-dried spinach also decreased colonic cytotoxicity and colonic hyperproliferation induced by heme [49]. These protective effects are attributed to the chlorophyll content, which prevents heme degradation and blocks the formation of cytotoxic heme metabolites [49].

Aside from skin, liver, stomach, and colon cancer, SCC has been reported to decrease the proliferation of human pancreatic cancer cell lines in vitro [70] and delay the progression of lung cancer in vitro and in vivo [71].

The anticarcinogenic activity of chlorophylls and SCC is suggested to occur through various mechanisms, including the formation of a molecular complex with aromatic carcinogens. This complex formation reduces carcinogen uptake and bioavailability, enhances elimination of the unmetabolized carcinogen, inhibits carcinogen activation, and promotes antioxidant activity and induction of apoptosis in cancer cells [2,51,72]. The protective effect through complex formation mainly occurs when chlorophylls are administered simultaneously with the carcinogen, highlighting the importance of consuming green and leafy vegetables and fruits to combat dietary carcinogens and mutagens [72]. Moreover, high-fiber diets have been associated with better prognosis in oncology [73], emphasizing the need for targeted policies to promote the consumption of healthier foods over processed ones, thereby improving public health.

Photodynamic therapy (PDT) is a two-stage treatment involving a photosensitizing drug and activating light, combined with molecular oxygen to induce cell death (phototoxicity). PDT has been increasingly used in cancer treatment [74]. However, the prolonged half-life of photosensitizers often leads to prolonged generalized cutaneous photosensitivity, causing a phototoxic reaction when the treated lesion is exposed to sunlight. Chlorophyll acts as a photosensitizer due to its natural ability to absorb light. Notably, it loses its photosensitizing activity within a few hours and requires only a relatively short incubation period [75], making it a promising candidate for PDT. In a clinical trial, cancer patients with basal cell carcinoma, squamous cell cancer, and papillary carcinoma received a single intravenous injection of mono-L-aspartyl chlorin e6 (NPe6), a photosensitizer derived from chlorophyll a. The study showed that NPe6 PDT had some efficacy against cutaneous tumors with minimal phototoxic side effects [76]. Previous research demonstrated that NPe6 persisted in the plasma of cancer patients for 6 weeks, although no persistent skin photosensitization was observed [77]. NPe6 PDT has also been used for the treatment of lung cancer [74]. Recently, Zhuo et al. [78] demonstrated that chlorophyllin e6-mediated PDT induces apoptosis in human bladder cancer cells, possibly through the inhibition of superoxide dismutase activity and the generation of reactive oxygen species. Although the use of chlorophyll or NPe6 in PDT shows promising results, further clinical trials with robust and compelling positive outcomes are needed to confirm their efficacy.

#### 3.3.4. Anti-Obesity Effects

The prevalence of obesity has significantly increased in recent decades, becoming a major concern in developed countries. The imbalance between energy intake and energy expenditure, coupled with reduced physical activity, is one of the primary factors contributing to the development of obesity [79]. To combat obesity and its associated diseases such as diabetes, cardiovascular diseases, and atherosclerosis [80], it is crucial to make dietary changes that prioritize the consumption of healthier foods. Recent studies have suggested that chlorophylls could have a positive impact on obesity control.

In an in vitro study, chlorophyll a extracted from the aquatic plant *Ludwigia octovalvis* exhibited an antiproliferative effect on 3T3-L1 adipose cells. It induced cell apoptosis by activating the CD95 (APO/CD95) death receptor and pro-caspase-3 proteins. The anti-adipogenic activity of chlorophyll a appeared to be mediated through the activation of the AMPK signaling transduction pathway [81]. Another study by Wang et al. [82] investigated the effects of chlorophyll and its derivatives on the digestion of soybean oil under simulated human gastrointestinal conditions. The researchers discovered that chlorophyll reduced the release rate of free fatty acids, altered the fatty acid composition, and increased the particle size of oil droplets. These changes could decrease the uptake of fatty acids by intestinal epithelial cells. Furthermore, the study found that pheophytin bound to and inhibited pancreatic lipase activity during intestinal digestion, thereby affecting lipid digestion in vitro [82].

Thylakoids, which are membranes within plant chloroplasts where photosynthesis occurs, consist of galactolipids, proteins, vitamins, antioxidants, chlorophylls, carotenoids, and other pigments [83]. Spinach baby leaves, for example, may contain around 3000 mg of chlorophyll per 100 g of thylakoids [84]. In vivo studies have demonstrated that thylakoid supplementation can suppress appetite, reduce body weight gain and body fat, lower serum glucose, triglyceride, and free fatty acid levels, and modulate appetite-regulating hormones in animals fed a high-fat diet (HFD) [85,86]. Supplementation with thylakoids has also been shown to induce weight loss, decrease total and LDL cholesterol levels, suppress the appetite for palatable food in overweight women [84], and exert a prebiotic effect that influences microbiota composition, thus improving lipid and glucose homeostasis [83].

A study by Seo et al. [87] investigated the anti-obesity and browning effects of *Spirulina maxima* extract, a microalga rich in chlorophyll a. The researchers found that Spirulina extract suppressed lipid accumulation by reducing the expression of adipogenic and lipogenic proteins *in vitro*. Furthermore, supplementation with Spirulina extract decreased body weight gain, fat mass, triglyceride and total cholesterol serum levels, and also reduced the expression of adipogenic proteins while increasing thermogenic factors in mice fed an HFD [87]. In the same study, chlorophyll a alone was found to inhibit adipogenesis and lipogenesis in vitro [87].

Other recent studies reported that supplementation with chlorophyll-rich spinach extract significantly reduced body weight gain, low-grade inflammation, and improved glucose tolerance in mice fed an HFD [88,89]. Moreover, this chlorophyll-rich extract supplementation also alleviated gut microbiota dysbiosis induced by the HFD [88,89]. Additional studies have demonstrated that chlorophyll-rich thylakoid supplementation or chlorophyllin can modulate the diversity and composition of gut microbiota in human subjects and BALB/c mice, respectively [90,91]. The composition of the gut microbiota is now recognized to be related to the state of health or disease, including obesity [92]. It has been observed that dietary substances can influence the composition of the gut microbiota [93,94]. Furthermore, there is evidence of an interaction between herbal medicines and gut microbiota. It is suggested that the gut microbiota biotransforms components of herbal medicines into bioactive small molecules that are absorbed into the bloodstream. Simultaneously, herbal medicines can modify the composition of gut microbiota by promoting the growth of beneficial bacteria and inhibiting harmful ones, leading to favorable physiological changes [95].

#### 3.3.5. Protection against Endocrine Disruptors

Research suggests that chlorophyll has the ability to mitigate the harmful effects of endocrine disruptors through several mechanisms. Firstly, chlorophyll has been found to possess antioxidant properties. It can scavenge reactive oxygen species (ROS) and reduce oxidative stress, which is known to be associated with endocrine disruption. By reducing oxidative stress, chlorophyll helps to protect endocrine organs, such as the ovaries, testes, and thyroid gland, from damage caused by endocrine disruptors. Secondly, chlorophyll has been shown to exhibit detoxification properties. It can bind to and effectively remove certain endocrine-disrupting chemicals from the body. This binding ability, known as chelation, helps to prevent the chemicals from interacting with hormone receptors and disrupting normal endocrine function. By facilitating the elimination of these harmful compounds, chlorophyll aids in reducing their detrimental effects on the endocrine system [96,97]. A study from Okai et al. [98] showed that, compared to SCC, chlorophyll *a* and pheophytin *a* derived from green tea showed strong preventive effects against the endocrine disruptor *p*-nonylphenol (NP)-induced inhibition of cell growth and cellular respiration in yeast *Saccharomyces cerevisiae*. These protective effects of natural chlorophylls were linked to their ability to attenuate oxygen radical formation induced by NP in yeast cells.

Furthermore, chlorophyll has been found to possess anti-inflammatory properties. Endocrine disruptors can induce chronic inflammation, which can further exacerbate their disruptive effects on hormone regulation. Chlorophyll has been shown to modulate inflammatory pathways and reduce the production of inflammatory mediators, thereby mitigating the inflammatory response triggered by endocrine disruptors [99]. A water-extract from the leaves of the red algae Dulse, constituted by phycobiliproteins and chlorophyll *a*, attenuated the secretion of tumor necrosis factor-α (TNF-α), interleukin-6 (IL-6) and nitric oxide induced by LPS in vitro [100]. Furthermore, the acid-insoluble fraction of the water-extract, containing concentrated pheophorbide *a* and pheophorbide *a* derivatives, was able to reduce the secretion of proinflammatory mediators. Additionally, the oral administration of the Dulse water-extract to mice decreased the acute inflammation in carrageenan-induced paw edema [100]. Moreover, chlorophyll has been observed to support liver health and enhance detoxification processes. The liver plays a crucial role in metabolizing and eliminating endocrine-disrupting chemicals from the body. Chlorophyll promotes liver function and aids in the detoxification of these compounds, thereby reducing their accumulation and potential impact on the endocrine system. While more research is needed to fully understand the extent of chlorophyll’s protective effects against endocrine disruptors, the available evidence suggests its potential as a natural defense mechanism. Incorporating chlorophyll-rich foods, such as leafy green vegetables, into the diet may contribute to minimizing the adverse effects of endocrine disruptors on hormone balance and overall health [51,101].

#### 3.3.6. Neuroprotective and Anti-Inflammatory Effects

One way chlorophyll may exert its neuroprotective effects is through its antioxidant properties. Oxidative stress, which occurs when there is an imbalance between the production of harmful free radicals and the body’s antioxidant defenses, has been implicated in the development and progression of neurodegenerative diseases such as Alzheimer’s, Parkinson’s, and Huntington’s diseases. Chlorophyll’s antioxidant activity allows it to neutralize free radicals and reduce oxidative damage to brain cells. By reducing oxidative stress, chlorophyll may help preserve the structure and function of neurons, potentially slowing down the onset or progression of neurodegenerative disorders [102]. Rehni et al. [103] demonstrated that a pre-treatment with a chlorophyll salt had neuroprotective effects in mice subjected to cerebral ischemia followed by reperfusion. Results showed that chlorophyll was able to decrease cerebral infarct size, increase short-term memory, attenuate motor incoordination and increase lateral push response. These protective effects were attributed to the antioxidant activity of chlorophylls. Additionally, chlorophyll has been shown to possess anti-inflammatory properties. Chronic inflammation is a common feature of many neurological conditions, and it can contribute to neuronal damage and degeneration. By modulating inflammatory pathways, chlorophyll may help dampen excessive inflammation in the brain, thereby protecting neurons from inflammatory damage. Furthermore, chlorophyll has been found to enhance the body’s natural detoxification processes. Toxins and environmental pollutants can accumulate in the brain and contribute to neuronal dysfunction and neurodegenerative processes [104,105]. Chlorophyll’s detoxifying properties may aid in the elimination of these harmful substances, reducing their impact on brain health and potentially preventing or delaying neurodegenerative conditions. Moreover, chlorophyll-rich foods have been associated with improved cognitive function and brain health. Leafy green vegetables, which are abundant sources of chlorophyll, have been linked to better cognitive performance and a lower risk of cognitive decline [47]. While the exact mechanisms underlying these associations are not fully understood, the neuroprotective properties of chlorophyll may play a role. It is important to note that the research on chlorophyll’s neuroprotective effects is still in its early stages, and more studies are needed to fully elucidate the extent and mechanisms of its action. Nonetheless, the available evidence suggests that incorporating chlorophyll-rich foods into the diet may contribute to brain health and potentially offer protection against neurodegenerative disorders. As always, maintaining a balanced and nutritious diet, along with a healthy lifestyle, is crucial for overall brain health and well-being [106].

In general, the bibliography from the last decades has shown essentially the potential of chlorophyll and chlorophyllin as anticancer and anti-obesogenic agents, but this evidence comes basically from a few in vitro and in vivo studies. The same applies for the other health benefits mentioned above. This lack of reliable clinical trials and follow-up studies in humans also translates into doubts regarding the real beneficial properties of chlorophylls. Nevertheless, these gaps should not divert attention from the potential use of these pigments, and these questions should continue to be addressed in future studies.

## 4. Chlorophyll Content in Fruits and Vegetables

Modern societies are currently facing food waste problems that are increasing as the world’s population also increases, leading to economic and environmental issues. Food losses and waste occur at all stages of the food supply chain: agricultural production, post-harvest handling and storage, processing, distribution, and consumption stages [4]. Simultaneously with these difficulties, changes in eating habits, increased consumption of more processed foods, and less variety in diets have contributed to the increase in modern societies’ diseases such as obesity, diabetes, cardiovascular diseases, and atherosclerosis. The discarded material can be a valuable resource to answer these problems. For instance, leafy material or fruit peels, which are discarded in these first stages, are usually rich in bioactive compounds beneficial to health [107,108,109]. The use of these currently discarded products may represent a return to past eating habits, with the use of more diverse foods, sometimes not so appealing, but with less caloric concentrations and rich in a high variety of bioactive compounds. For example, broccoli is one of the most produced crops worldwide, where only the inflorescence part is used, while the stem and leaves are discarded. Nevertheless, this discarded material, in addition to glucosinolates, is also extremely rich in chlorophylls, especially the leaves. Our group evaluated the chlorophyll and carotenoid contents in the broccoli plant in two different harvest years (Supplementary Methods). Table 1 shows the contents of chlorophylls *a* and *b*, total chlorophylls, and total carotenoids in the inflorescences, stalks and leaves of broccoli plants, harvested in October 2018 and July 2019. For both crops, the leaves are the part of the broccoli plant that contains significantly (*p* < 0.05) more total chlorophyll content and carotenoids compared to stems and inflorescences. Comparing the two harvests, only the leaves registered significant differences (*p* < 0.0001), whereas the 2018 crop plants showed higher contents of pigments than the 2019 crop. This difference may be due to the fact that plants harvested in July were subjected to greater environmental stress [110]. These results can also be correlated with the high antioxidant activity of the leaves compared to stalks and inflorescences [111]. These results suggest that the leaves can be a broccoli by-product with high interest and potential for exploitation as a functional food ingredient due to their high content of chlorophylls. Nevertheless, the stalks can also be a value-added product of great potential since chlorophylls are also present in high amounts.

When it comes to green plants and vegetables, storage and processing conditions greatly impact the green color of these foods conferred by chlorophyll, whose degradation can be delayed or accelerated by these conditions [112,113,114,115,116]. This, in turn, has a great influence on the behavior of the final consumer, that is, not consuming them if the products do not have an attractive green color, thus further contributing to food waste.

Table 2 lists chlorophyll-rich fruits and vegetables. Compared to fruits, vegetables are particularly rich in chlorophyll, among which spinach, broccoli, garden rocket and wild rocket have the highest amount of this pigment. On the contrary, fruits and vegetables that are lower in chlorophyll include: kiwifruit purée, cabbage, celery and cucumber. For some fruits and vegetables, the extent of chlorophyll loss after food processing is also indicated (Table 2). This is particularly important since several processing food techniques may have a large contribution to final chlorophyll content, affecting the nutritional and commercial value. The green color, conferred by a high proportion of chlorophylls *a* and *b*, is often used as a quality measurement [117]. The post-harvest storage time *per se* leads to the loss of green color due to chlorophyll degradation, triggered by ROS and ethylene formation [118]. The use of exogenous diacetyl to inhibit ethylene and ROS generation, has been proposed to retard the yellowing and nutrient loss of postharvest broccoli, thus maintaining its nutritional and economic value [119]. Postharvest treatment of broccoli with melatonin has also been shown to prolong shelf life [120]. Table 2 shows the chlorophyll content in fruits and vegetables before and after processing. However, this information has to take into account the concepts previously described regarding its bioavailability. Furthermore, recent data shows that food composition is also a variable that greatly influences the bioavailability of chlorophylls. Viera et al. [121] have shown that, during the in vitro digestion, the stability of total chlorophylls ranges from 15% to 85%, which is influenced by the amount of salt present in the food.

Based on the provided information, here are some observations regarding the processing methods and conditions that can help retain higher chlorophyll content in certain fruits and vegetables: (i) Boiling: Boiling for a shorter duration appears to be more effective in retaining chlorophyll content. For example, in the case of green beans, boiling for 5 min resulted in higher chlorophyll content compared to longer boiling times; (ii) Steaming: Steaming for a moderate duration seems to be beneficial for maintaining chlorophyll levels. In the case of spinach, steaming for 7.5 min resulted in higher chlorophyll content compared to both shorter and longer steaming times; (iii) Microwaving: Microwaving for a shorter duration tends to preserve chlorophyll content. For instance, in the case of peas, microwaving for 1.5 min resulted in higher chlorophyll content compared to longer microwaving times; (iv) Storage conditions: Some vegetables, such as celery and leek, demonstrated a decrease in chlorophyll content after storage at low temperatures (0 °C) for an extended period. Therefore, it is advisable to minimize storage time at low temperatures to maintain higher chlorophyll levels.

It is important to note that the optimal method and conditions for preserving chlorophyll content may vary depending on the specific fruit or vegetable being processed. Additionally, other factors such as the desired texture, taste, and nutrient retention should also be considered when determining the best processing method for a particular food item. Further research and experimentation may be necessary to obtain more specific and comprehensive guidelines for maximizing chlorophyll retention during food processing.

## 5. Conclusions and Future Perspective

The rise in obesity, diabetes, cardiovascular diseases, and atherosclerosis can be attributed to significant changes in human dietary patterns. However, the precise pharmacokinetics of dietary chlorophylls and their derivatives, which play a crucial role in conferring health benefits, remain poorly understood, needing further investigation. While native chlorophylls have been the focus of limited research, both in vitro and in vivo studies have shed light on the therapeutic potential of chlorophyll derivatives. These derivatives exhibit a range of beneficial effects, including antioxidant, antimutagenic, antigenotoxic, anticarcinogenic, and anti-obesogenic properties. Nevertheless, additional research is required to validate the efficacy of dietary chlorophylls in treating the aforementioned diseases and to explore their therapeutic potential in other medical conditions.

It is well-established that a high intake of vegetables and fruits promotes improved health outcomes. The synergistic interplay among various bioactive compounds, rather than the action of a single component, primarily underlies these benefits. In light of this, investigating the potential health advantages and circular economy value of discarded agri-food materials, such as broccoli leaves, which contain chlorophyll and other phytochemicals, presents an intriguing avenue for research. Such exploration can provide valuable insights into harnessing the health-promoting properties of these wasted resources and their potential contribution to a sustainable and holistic approach to nutrition.

## Figures and Tables

**Table 1:** Contents of chlorophyll *a*, chlorophyll *b*, total chlorophylls, and total carotenoids (mg/g DW), in the inflorescences, stalks and leaves of broccoli plants.

	Chlorophyll *a*		Chlorophyll *b*		Total Chlorophylls		Total Carotenoids	
Broccoli Part	2018	2019	2018	2019	2018	2019	2018	2019
Inflorescence	0.75 ± 0.05 **^aA^**	0.91 ± 0.09 **^bA^**	0.30 ± 0.03 **^aA^**	0.34 ± 0.03 **^aA^**	1.05 ± 0.24 **^bA^**	1.25 ± 0.24 **^bA^**	0.18 ± 0.02 **^bA^**	0.26 ± 0.02 **^bA^**
Stalk	0.30 ± 0.03 **^aA^**	0.14 ± 0.03 **^aA^**	0.14 ± 0.01 **^aA^**	0.05 ± 0.01 **^aA^**	0.44 ± 0.12 **^bA^**	0.19 ± 0.081 **^cA^**	0.07 ± 0.004 **^aA^**	0.05 ± 0.01 **^aA^**
Leaves	12.43 ± 0.14 **^bA^**	4.96 ± 0.36 **^cB^**	4.74 ± 0.06 **^bA^**	1.85 ± 0.16 **^bB^**	17.17 ± 0.59 **^aA^**	6.81 ± 0.36 **^aB^**	2.49 ± 0.03 **^cA^**	1.15 ± 0.08 **^cB^**

Data presented as mean ± SD (*n* = 5). For each year, values for the same parameter evaluated (within the same column) followed by different lowercase letters are significantly different at *p* < 0.05, according to Tukey’s test. Uppercase letters indicate differences between the harvest years (*p* < 0.0001). DW, dry weight.

**Table 2:** Chlorophyll content in fruits and vegetables before and after processing.

Fruit/Vegetable	Total Chlorophyll Content (Fresh/Raw Material)	Food Processing	Chlorophyll Content after Processing	References
**Broccoli**	19.1 mg/kg (d.w.)			[122]
	21 mg/kg (f.w.)			[123]
	72.6 mg/kg (f.w.)			[124]
	128.2 mg/kg (f.w.)			[112]
	6940 mg/kg (d.w.)	Boiling for 5 min	4140 mg/kg (d.w.)	[115]
		Steaming for 7.5 min	2800 mg/kg (d.w.)	
		Microwaving for 1.5 min	4100 mg/kg (d.w.)	
**Brussels sprouts**	31.8 mg/kg (f.w.)			[125]
	57.5 mg/kg (f.w.)	Microwaving for 6 min	30.4 mg/kg (f.w.)	[124]
**Cabbage**	15.3 mg/kg (f.w.)			[124]
**Celery**	23 mg/kg (f.w.)			[123]
	34.5 mg/kg (f.w.)	Stored at 0 °C for 21 days	15 mg/kg (f.w.)	[116]
		Thermally treated by immersion (50 °C for 90 s) and stored at 0 °C for 21 days	30 mg/kg (f.w.)	
		Thermally treated by heated air (48 °C for 1 h) and stored at 0 °C for 21 days	14 mg/kg (f.w.)	
**Cucumber**	36 mg/kg (f.w.)			[123]
**Dandelion**	2482.5 mg/kg (f.w.)			[126]
**Garden rocket**	3596.2 mg/kg (f.w.)			[126]
**Green beans**	71–133 mg/kg (f.w.)			[127]
	75 mg/kg (f.w.)			[123]
	1850 mg/kg (d.w.)	Boiling for 5 min	1040 mg/kg (d.w.)	[115]
		Steaming for 7.5 min	960 mg/kg (d.w.)	
		Microwaving for 1.5 min	1110 mg/kg (d.w.)	
**Green paprika**	38 mg/kg (f.w.)			[123]
**Green peas**	50 mg/kg (f.w.)			[123]
**Green pepper**	86.1 mg/kg (f.w.)			[112]
	797.8 mg/kg (d.w.)			[122]
**Kale**	282 mg/kg (f.w.)	Heat steam sterilization at 121 °C for 5 min	n.d.	[128]
	1834 mg/kg (f.w.)	Microwaving for 6 min	1142 mg/kg (f.w.)	[124]
**Kiwifruit purée**	6.8 mg/kg (f.w.)	Puréed fruit after boiling for 5 min	n.d.	[129]
		Frozen purée at −18 °C stored for:		
		1 day	3.9 mg/kg (f.w.)	
		36 days	1.9 mg/kg (f.w.)	
		68 days	0.9 mg/kg (f.w.)	
**Leek**	1800 mg/kg (d.w.)	Boiling for 5 min	480 mg/kg (d.w.)	[115]
		Steaming for 7.5 min	530 mg/kg (d.w.)	
		Microwaving for 1.5 min	710 mg/kg (d.w.)	
**Lettuce**	2888.1 mg/kg (d.w.)			[122]
**Parsley**	632 mg/kg (f.w.)			[123]
	995 mg/kg (f.w.)	Heat steam sterilization at 121 °C for 5 min	n.d.	[128]
**Peas**	87 mg/kg (f.w.)			[123]
	1310 mg/kg (d.w.)	Boiling for 5 min	1170 mg/kg (d.w.)	[115]
		Steaming for 7.5 min	1200 mg/kg (d.w.)	
		Microwaving for 1.5 min	1210 mg/kg (d.w.)	
**Spinach**	639.1 mg/kg (f.w.) (spinach purée)			[128]
	791 mg/kg (f.w.)			[123]
	1083.4 mg/kg (f.w.)			[112]
	1148 mg/kg (f.w.)			[124]
	9470 mg/kg (d.w.)	Blanched	9250 mg/kg (d.w.)	[113]
		Processed at 121 °C for:		
		4 min	6800 mg/kg (d.w.)	
		15 min	1480 mg/kg (d.w.)	
		30 min	n.d.	
	20330 mg/kg (d.w.)	Baking at 105 °C for:		[114]
		20 min	14000 mg/kg (d.w.)	
		40 min	9580 mg/kg (d.w.)	
		80 min	7380 mg/kg (d.w.)	
		Blanching for:		
		6 min	9670 mg/kg (d.w.)	
		9 min	8270 mg/kg (d.w.)	
		15 min	5270 mg/kg (d.w.)	
		Steaming for:		
		7.5 min	4990 mg/kg (d.w.)	
		30 min	3510 mg/kg (d.w.)	
		60 min	1140 mg/kg (d.w.)	
		Microwave cooking for:		
		1 min	16,770 mg/kg (d.w.)	
		5 min	11,850 mg/kg (d.w.)	
		9 min	7980 mg/kg (d.w.)	
	39,090 mg/kg (d.w.)	Boiling for 5 min	25,770 mg/kg (d.w.)	[115]
		Steaming for 7.5 min	25,380 mg/kg (d.w.)	
		Microwaving for 1 min	24,740 mg/kg (d.w.)	
**Squash**	1660 mg/kg (d.w.)	Boiling for 5 min	890 mg/kg (d.w.)	[115]
		Steaming for 7.5 min	770 mg/kg (d.w.)	
		Microwaving for 1.5 min	680 mg/kg (d.w.)	
**Wild rocket**	3032.3 mg/kg (f.w.)			[126]
**Zucchini**	68 mg/kg (f.w.)			[123]

f.w., fresh weight; d.w., dry weight; n.d., not detectable.
